# Testing the Efficacy of Global Biodiversity Hotspots for Insect Conservation: The Case of South African Katydids

**DOI:** 10.1371/journal.pone.0160630

**Published:** 2016-09-15

**Authors:** Corinna S. Bazelet, Aileen C. Thompson, Piotr Naskrecki

**Affiliations:** 1 Department of Conservation Ecology and Entomology, Stellenbosch University, Private Bag X1, Matieland, 7602, South Africa; 2 Museum of Comparative Zoology, Harvard University, Cambridge, Massachusetts, 02138, United States of America; University of Colorado, UNITED STATES

## Abstract

The use of endemism and vascular plants only for biodiversity hotspot delineation has long been contested. Few studies have focused on the efficacy of global biodiversity hotspots for the conservation of insects, an important, abundant, and often ignored component of biodiversity. We aimed to test five alternative diversity measures for hotspot delineation and examine the efficacy of biodiversity hotspots for conserving a non-typical target organism, South African katydids. Using a 1° fishnet grid, we delineated katydid hotspots in two ways: (1) count-based: grid cells in the top 10% of total, endemic, threatened and/or sensitive species richness; vs. (2) score-based: grid cells with a mean value in the top 10% on a scoring system which scored each species on the basis of its IUCN Red List threat status, distribution, mobility and trophic level. We then compared katydid hotspots with each other and with recognized biodiversity hotspots. Grid cells within biodiversity hotspots had significantly higher count-based and score-based diversity than non-hotspot grid cells. There was a significant association between the three types of hotspots. Of the count-based measures, endemic species richness was the best surrogate for the others. However, the score-based measure out-performed all count-based diversity measures. Species richness was the least successful surrogate of all. The strong performance of the score-based method for hotspot prediction emphasizes the importance of including species’ natural history information for conservation decision-making, and is easily adaptable to other organisms. Furthermore, these results add empirical support for the efficacy of biodiversity hotspots in conserving non-target organisms.

## Introduction

Global biodiversity hotspots are regions with exceptionally high levels of plant endemism that are threatened by high rates of habitat loss [1]. Although no animal data were used to delineate these hotspots, they are also known to contain high levels of vertebrate endemism. While the current definition relies on endemic species as a surrogate because they have limited geographic ranges and are therefore more vulnerable to extinction, Myers [2] argues that other criteria, such as species richness, rarity, and taxonomically unusual species, could be employed to achieve the same outcome. Historically, species richness was used more often for a variety of conservation prioritization purposes than endemism since these data are more readily available and, intuitively, the more species a region contains, the more worthy it is of conservation [3, 4]. However, assessing species richness alone without any sense of the composition of the species means that rare or sensitive species may be overlooked [3]. This has led to the development of a variety of alternative methods for assessing conservation priority among regions.

The simplest method for taking species composition into account in the selection of regions of conservation priority is by calculating species richness of certain target taxa only, such as the threatened or endemic species alone, rather than species richness as a whole. For birds, it has been shown that there exists little congruence between hotspots of endemism, threat and species richness [5, 6]. Global patterns of species richness and endemism are highly correlated among taxa for amphibians, reptiles, birds and mammals, but are not concordant within taxa [7]. North American mammal and insect species richness and endemism, on the other hand, are correlated within taxa but differ greatly among taxa [8]. In the absence of fine-scale information, areas with high levels of endemism are expected to protect not only those endemic organisms for which they were selected, but also a large diversity of organisms in general, making endemism the most widely agreed upon surrogate measure for hotspot identification [6].

While endemism is a descriptor of one element of a species’ biology, most assessment techniques are still constructed on the basis of a count of species. Several measures have gone one step beyond simply counting species, to giving species a weighted score on the basis of some aspect of their biology. Weighted endemism, which assigns weights to species on the basis of their geographic range such that smaller ranges score higher, is an alternate approach to simply selecting a binary definition of endemism and counting species which fall below the threshold [9, 10]. Similarly, phylogenetic diversity scores species on the basis of their evolutionary history, and gives higher weights to regions which are more phylogenetically diverse and distinct, and can be applied together with measures of spatial rarity for more robust conservation planning [10, 11].

New methods for rapid assessment and ranking of habitats hold some potential for extrapolation to larger spatial and temporal scales and assessment of regional, national or global diversity patterns. The Dragonfly Biotic Index (DBI) is one such index which is used to assess ecological integrity of freshwater habitats in South Africa [12, 13]. This weighted assessment technique has proven to be successful because dragonflies have a close association with riparian vegetation and are observably impacted by changes (positive or negative) to their habitats [14, 15]. There is also a great deal of biological information available regarding South Africa’s dragonfly communities, enabling each species to be assigned rankings on various traits. These rankings can be compared among individual species or averaged across all species occurring in a specific habitat in order to assign a score to the habitat as a whole and enabling the comparison of different habitats on the basis of their dragonfly assemblage.

South Africa contains three recognized global biodiversity hotspots: Succulent Karoo, Cape Floristic Region (CFR), and Maputaland-Pondoland-Albany (MPA) [1, 16]. These hotspots, like all global hotspots, were selected for having high plant endemism and high levels of threat, irrespective of any animal data, although high levels of vertebrate endemism were also detected in these regions. Although invertebrates were omitted from original assessments which justified the delineation of these hotspots, Myers *et al*. (2000) suggested that, on the basis of sheer number of unique plant-insect interactions that exist within these hotspots, diversity of insects is expected to mirror that of the endemic plants. The CFR, in particular, has been the focus of much debate regarding whether insect diversity does, in fact, mirror that of the plants [17–20]. For some insect groups, particularly the gall-forming insects [21, 22] and the leafhoppers [23, 24] insect diversity does appear to mirror that of plants, while for others like ants [25] and butterflies [26], insect diversity is much lower than plant diversity.

South African katydids (or bush crickets; Orthoptera: Tettigonioidea) are a charismatic, nocturnal group of insects which range from small-bodied, monophagous herbivores to voracious predators which are among the largest of the insects in their habitats [27]. During the summer months, the males produce a species-specific call in order to attract a mate. South Africa contains several fascinating groups of resident katydids, particularly along the west coast in the CFR and Succulent Karoo biomes. Southern Africa hosts an endemic tribe, the Aprosphylini (Tettigoniidae: Mecopodinae) which appears to be a Gondwanaland relict [28]. This tribe contains the only known cave katydid in the world (*Cedarbergeniana imperfecta* Naskrecki, 1993), several species which, unusually for katydids, live beneath rocks (*Griffiniana* spp.) [29], and a specialized leaf litter katydid (*Zitsikama tessellata* Peringuey, 1916). There is also a species radiation of small, flightless, herbivorous katydids with a north-south distribution along South Africa’s west coast, *Brinckiella* spp. [30]. Little is known of katydid distribution patterns across South Africa, but recent Red Listing of the entire fauna employing extensive field surveys, historical museum records and species specific biological information, have made it possible to assess katydid distribution patterns across South Africa, and to compare count-based methods with scoring methods for identification of katydid hotspots.

In this study, we aim to define hotspots of katydid diversity in South Africa, Lesotho and Swaziland (referred to as South Africa for simplification throughout) and assess whether they are congruent with global biodiversity hotspots. To do this, we first develop a species scoring system which utilizes knowledge about each species’ IUCN Red List threat status, distribution, mobility and trophic level. To validate our species scoring system, we first examine the covariation of species traits and their distribution across taxa. We then define katydid hotspots in two ways: by using a species richness count approach vs. a species composition scoring approach. Finally, we compare our two types of katydid hotspots with each other and with South Africa’s recognized biodiversity hotspots in order to draw conclusions about katydid diversity and distribution across South Africa, and the implications of taking species’ biological traits into account when assessing the efficacy of global biodiversity hotspots for the conservation of non-traditional target organisms.

## Methods

### Katydid Red Listing

Over two decades, PN visited global museum collections, identified specimens and recorded locality data and measurements into his MANTIS database [31]. Using MANTIS and OSF [32], a list of 167 katydid species known to occur in South Africa, Lesotho and Swaziland was compiled. Of the full list, 133 species (79.64%) were assessed for the IUCN’s Red List [33]. Taxa which could not be assessed (n = 34; 20.35%) included members of large genera in great need of scientific revision (e.g. *Ruspolia* spp.) and subspecies of questionable validity (e.g. *Hetrodes pupus* subspp.).

For Red List assessment, CSB first calculated extent of occurrence (EOO) and area of occupancy (AOO) in ArcGIS 9.2 [34] on the basis of collection records stored in MANTIS. Species were then assessed in accordance with IUCN assessment criteria [35] using either Criterion B (geographic range in the form of EOO and/or AOO) or Criterion D (very small or restricted population) into one of six statuses: Critically Endangered (CR), Endangered (EN), Vulnerable (VU), Least Concern (LC), or Data Deficient (DD). Assessment text was written by CSB and PN and all assessments were published by the IUCN in 2014 [33]. DD species (n = 16) were excluded from further analyses.

### Katydid scoring and diversity measures

Each species was scored for several traits (Table 1). Threat status (T) was scored a value between 0–3 in ascending order of threat. Distribution (D) was scored from 0–3 by decreasing distribution range size (the narrower the species’ range, the higher its score). Life history (LH) was scored as the sum of two separate scores: mobility (M) was scored from 0–2 in descending order of mobility (e.g. 2 = flightless) and trophic level (Tr) was scored from 0–3 in ascending order of food specialization (e.g. 3 = single host herbivore). Combinations of these elements were summed and their spatial distribution mapped. When all elements were summed, the total maximum score was 9, and the higher this value, the more threatened, endemic, and host specialized the species. This scoring system is similar to the Dragonfly Biotic Index [12, 13] and allows for species traits to be taken into account in diversity analyses. Since species scores were integers which ranged from 0–9, their residuals were not normally distributed (Shapiro Wilk’s W = 0.96, p = 0.001) so species traits were compared among threat categories using Kruskal-Wallis nonparametric tests in R 3.0.2 [36] and Tukey-Kramer-Nemenyi post-hoc tests in package PMCMR [37].

**Table 1:** South African katydid scoring chart to enable comparison of species on the basis of three criteria: threat, distribution and life history traits.

Species Score	Threat (T)	Distribution (D)	Life History Traits (LH)^†^		
			*Mobility (M)*	*Trophic Level (Tr)*	*M+Tr Sum*
0	LC	Very common: *> 75% coverage of SA and sA*	*Fully-flighted*	*Omnivorous*	0
1	VU	Localized across a wide area in SA, and localized or common in sA: *> 66% in SA and > 66% sA*	*Only one sex flighted*	*Predatory*	1–2
		-OR-	*-OR-*		
		Very common in 1–3 provinces of SA and localized or common in sA: *0–33% SA and > 66% sA*	*One or both sexes partially flighted*		
2	EN	National SA endemic confined to 3 or more provinces: *> 33% SA*	*Flightless*	*Herbivorous*, *polyphagous*	3
		-OR-			
		Widespread in sA but marginal and very rare in SA: *< 33% SA and > 66% sA*			
3	CR	Endemic or near-endemic and confined to only 1 or 2 SA provinces: *< 33% in SA alone*		*Herbivorous*, *monophagous*	4–5

Each of the three categories is scored from 0 to 3, and the categories can be summed in different combinations to give each katydid species a score ranging from 0 to 9, with the higher the score, the more threatened, narrowly distributed, and specialized the katydid species. Threat scores are given in accordance with IUCN Red List categories and distribution scores are indicative of the number of countries (southern Africa) and provinces (South Africa) in which the species is found. Life history scores are awarded on the basis of a species’ mobility and its trophic level. SA = South Africa, Lesotho, and Swaziland and sA = southern Africa (South Africa, Lesotho, Swaziland, Namibia, Botswana and Zimbabwe).

^†^ To calculate LH score, M (range 0–2) + Tr (range 0–3) are summed. The sum is assigned a logical species score (range 0–3).

### Mapping

South Africa was divided into equal sized grid squares of 1° longitude by 1° latitude in QGIS [38]. This grid cell size divided South Africa into 150 cells, 28 (19%) of which did not contain any katydid collection points. While this is a very coarse scale division, it was the most appropriate for this study because it has been used for similar studies on a global scale for birds [6] and due to the relatively low number of total collecting records in South Africa (N = 1075 records of LC, VU, EN and CR species; S1 Table), this division of South Africa resulted in an average of 8.81 ± 0.31 (s.e.) species per grid cell. If we had used smaller grid cells, there would necessarily be fewer collection points per grid cell, compromising the possible analyses of the data. Grid cells were clipped to the coastline, and land area within a grid cell was taken into account in analyses to account for variation in size of cropped grid cells.

Several metrics were calculated per grid cell: total, threatened (number of CR, EN and VU species), and sensitive species richness (number of species with LH score = 3). Endemic species richness was calculated as the number of species in a cell which had EOO < 5000 km^2^. This criteria was selected for three reasons: (1) in the IUCN Red List Criterion B, this is the cut-off for a species to be classified as EN; (2) 25.44% of species (29 species all of which are threatened) were included in this classification which is similar to the 25% of species cut-off used by similar studies [6]; and (3) there is a natural break in the dataset in that, at EOO < 5000 km^2^, there are much larger gaps between successive EOO values than at EOO > 5000 km^2^ (S1 Fig).

Six combinations of the katydid species trait scores were also averaged per grid cell: threat + distribution (T+D); threat + life history (T+LH); distribution + life history (D+LH); threat + distribution + mobility (T+D+M); threat + distribution + trophic level (T+D+Tr); threat + distribution + life history (T+D+LH). The scores for all species present in a grid cell were averaged to give each grid cell a mean value per metric.

### Statistical analysis

#### By species analysis

We tested for covariance among the species score components by using a phylogenetic least squares analysis (PGLS) in R 3.0.2 [39]. Our data points violated the assumption of independence necessary for linear regression models since we assumed that more closely related species would be more similar in terms of their threat, distribution, and life history traits. In PGLS we first constructed a phylogenetic tree to the species (S2 Fig). Higher taxon (subfamily) relationships were determined according to Mugleston *et al*. (2013) [40]. For paraphyletic subfamilies (Tettigoniinae, Pseudophyllinae, Mecopodinae and Meconematinae) we did the following: because no subfamily in our study was represented by > 20 species and because all of the representatives in our study appeared similar morphologically, in terms of their tribal assignment, and in terms of their South African distribution, we considered them monophyletic for the purposes of this study. They were placed on the branch of the tree from Mugleston *et al*. (2013) which corresponded to their closest relative. Since we lacked information on evolutionary relationships within subfamilies, genera and subgenera were assumed to be monophyletic. All species within a subgenus were assigned equal branch lengths, subgenera within a genus were assigned equal branch lengths, and all genera within a subfamily were also assigned equal branch lengths, such that two species from the same subgenus were considered more closely related evolutionarily than two species from different subgenera within the same genus, but no further ranking was assigned at species, subgenus or genus level. All branch lengths were kept equal to one to construct a conservative tree, and the tree was unrooted. The only species which may fall significantly in the wrong place is a Pseudophyllinae species from the coastal forests of the Eastern Cape which has yet to be described, and which appears to be of a different evolutionary origin than other South African members of this subfamily. Within the genus *Brinckiella*, evolutionary relationships between species pairs *B*. *wilsoni–B*. *arboricola* and *B*. *karooensis–B*. *mauerbergerorum* were assumed on the basis of recent morphological evidence [30].

In PGLS we constructed a series of models to test the relationship of T (dependent variable) with D, LH, M, Tr and their interaction terms (independent variables), and D (dependent) with LH (independent). Ordinary least square models (OLS) and phylogenetic equivalents (PGLS) were constructed for each pair of variables and their strength was compared using Akaike Information Criteria (AIC) to select the best performing model [41]. PGLS models also produced an estimate of phylogenetic covariance (λ), which indicated the strength of the phylogenetic effect [39].

#### By grid cell analysis

In order to compare the information provided by each of the diversity measures per grid cell, we constructed a spatial generalized linear mixed effects model (GLMM) in R 3.0.2. We could not calculate traditional pair-wise correlations between the diversity measures because we expected a large degree of spatial autocorrelation which would violate the assumption of independence among the data points (grid cells). We first calculated the degree of spatial autocorrelation in fitted general linear models (function glm in R 3.0.2) of each pair of diversity measures [42]. Moran’s I was calculated using package ncf in R [43]. We then calculated GLMM using the function glmmPQL in package MASS [44] by using Poisson errors with predictor diversity measure and land area within a grid cell as fixed effects and spatial structure modeled as an exponential correlation structure [6, 42]. Estimates of model fit were calculated using marginal r^2^ since this is appropriate for models with no random effects [45]. Here, we present results for species richness based diversity measures and for the T+D+LH diversity measure which takes species identity into account. Other combinations of katydid species trait scores are excluded because they are collinear with T+D+LH since they are constructed from individual elements of the full measure.

We then compared overlap of katydid hotspots with South African biodiversity hotspots. We first classified the grid cells according to whether they fell within a biodiversity hotspot or not. We tested four inclusion rules: a grid cell was considered to be within a biodiversity hotspot if > 25% (N = 62, 50.8% of cells), > 50% (N = 57, 46.7% of cells), > 75% (N = 47, 38.5% of cells), or 100% (N = 39, 32.0% of cells) of the area of the cell fell within a biodiversity hotspot. There was no significant difference between the four possible inclusion rules in the difference between the hotspot minus non-hotspot values for any of the diversity measures (Kruskal-Wallis χ^2^_3_ = 0.22, p = 0.98). Therefore, we chose to use 50% inclusion throughout all analyses as this is conservative but includes enough grid cells to allow for more robust analyses.

All three of the biodiversity hotspots are located along South Africa’s coastline. Sampling density was higher along coastlines (i.e. in the hotspots) than in South Africa’s interior. However, since much of our raw data were derived from historical museum records, it was impossible to know whether this was due to increased sampling along the coastlines due to easier access or whether more specimens were collected along the coastlines because there were more specimens along the coastlines. We compared whether sampling effort was equivalent and sufficient between the hotspot and non-hotspot grid cells using species accumulation curves (SACs) calculated in EstimateS [46]. Hotspot and non-hotspot grid cells were compared for each of the diversity measures using Mann-Whitney non-parametric tests in R 3.0.2.

Frequency histograms were constructed to identify a usable definition of katydid count-based and score-based hotspots. We then ran a series of chi-squared tests in R 3.0.2 to test whether individual grid cells which fell within a katydid count or score-based hotspot were more likely to also fall within a biodiversity hotspot than what would be predicted on the basis of chance alone.

## Results

Of a total of 133 katydid species whose Red List status could be assessed, 16 (12.0%) were assessed as DD and excluded from all further analyses. Seventy-six (57.1%) were LC, 17 (12.8%) were VU, 10 (7.5%) were EN and 14 (10.5%) were CR (S2 Table).

**Fig 1:**
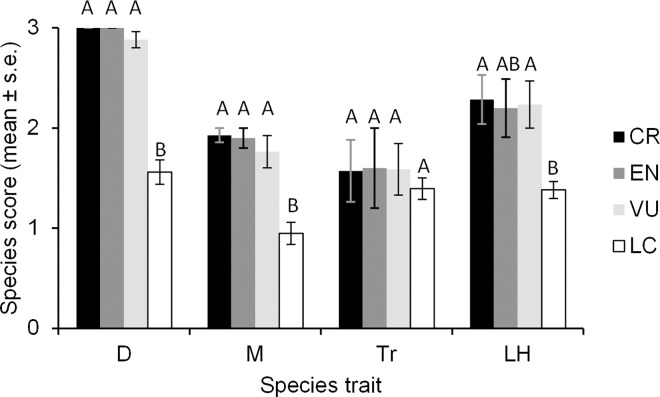
Bar graph illustrating trait differences among the four categories of Red Listed species. Capital letters indicate significant differences from a Tukey-Kramer-Nemenyi post-hoc test conducted following a Kruskal-Wallis global test. CR = Critically Endangered, EN = Endangered, VU = Vulnerable; LC = Least Concern. D = Distribution score, M = Mobility, Tr = Trophic level, LH = Life History.

LC species had significantly lower distribution, mobility and life history scores than CR, EN and VU species in almost all cases (Kruskal Wallis χ^2^_1_ = 56.84, p < 0.001; χ^2^_1_ = 25.00, p < 0.001; χ^2^_1_ = 23.89, p < 0.001, respectively; Fig 1). The three threatened categories did not differ from each other in any of the species traits.

The PGLS analysis showed that the best performing model described the relationship between distribution and life history with phylogeny taken into account (PGLS; Table 2). This model had a very strong phylogenetic signal, showing that more closely related species had a more similar distribution to life history relationship than distantly related species. The model which best explained a species’ threat status was the interaction term of distribution and life history followed by the interaction term of distribution and mobility. Phylogeny was not influential in any models where the dependent variable was threat status, indicating that threatened species are evenly distributed across subfamilies (Table 2).

**Table 2:** Ranked results of phylogenetic least squares analysis predictive models.

Rank	Dep		Ind1	Ind2	Model	AIC	λ
1	D	~	LH		PGLS	267.25	0.94
2	T	~	D	LH	OLS	307.96	
3	T	~	D	M	OLS	308.78	
4	T	~	D		OLS	309.86	
5	T	~	D	LH	PGLS	309.96	0.00
6	T	~	D	M	PGLS	310.78	0.00
7	T	~	D	Tr	OLS	311.32	
8	T	~	D		PGLS	311.86	0.00
9	T	~	D	Tr	PGLS	313.32	0.00
10	D	~	LH		OLS	323.14	
11	T	~	LH		PGLS	325.64	0.31
12	T	~	M		OLS	326.22	
13	T	~	M		PGLS	328.22	0.00
14	T	~	LH		OLS	329.13	
15	T	~	Tr		PGLS	338.85	0.53
16	T	~	Tr		OLS	349.35	

T = Red List threat status, D = distribution, M = mobility, Tr = Trophic level, and LH = life history (score based on combination of mobility and trophic level; see Table 1). OLS = Ordinary Least Squares, PGLS = Phylogenetic Least Squares; Dep = dependent variables, Ind = Independent variables, AIC = Akaike Information Criteria. λ = estimate of phylogenetic effect on model, value varies from 0–1 and the higher the value, the stronger the phylogenetic signal.

### Hotspot comparison

**Fig 2:**
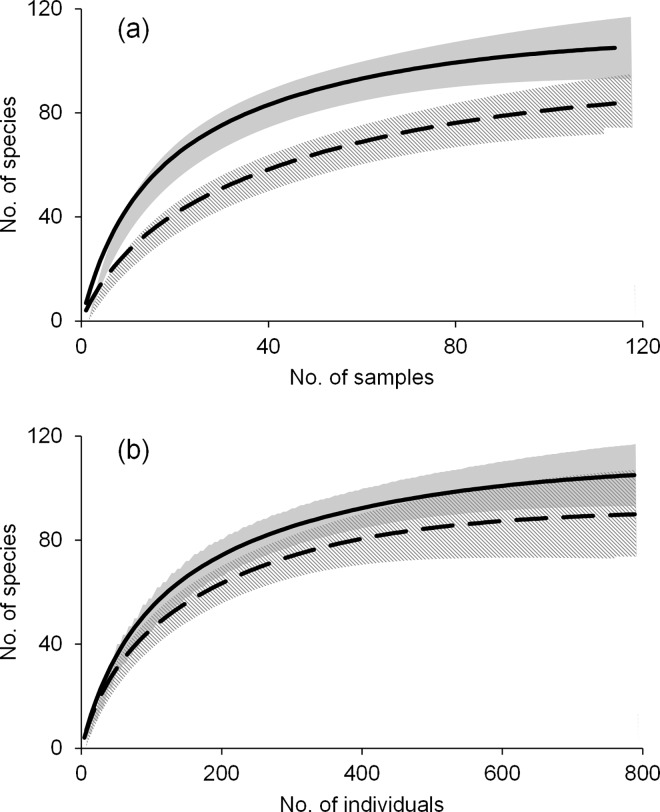
Species accumulation curves. Sample-based (a) and individual-based (b) species accumulation curves illustrating sufficiency of sampling of hotspot and non-hotspot grid cells. Shading indicates 95% confidence intervals. Hotspot grid cells = solid line and gray shading; Non-hotspot = dashed line and hashed shading.

Sample-based and individual-based SACs both showed that sampling was sufficient in hotspot and non-hotspot grid cells (Fig 2). The sample-based SAC had no overlap in confidence intervals, indicating that any differences in species richness between hotspot and non-hotspot grid cells was indicative of an ecological difference and not an artifact of uneven sampling effort. However, the confidence intervals in the individual-based SAC did overlap, indicating that species diversity patterns in the two types of grid cells may be a result of unequal sampling (Fig 2).

Although Moran’s I values were relatively low for fitted glm models for each pair of diversity measures (range 0.020 to 0.108), values were statistically significant in all cases, indicating significant spatial autocorrelation (p < 0.05 in all cases; Table 3). Slope estimates describing the relationship between each pair of diversity measures were positive and high (range 0.182 to 0.686), and spatial GLMMs all showed a statistically significant relationship between each pair of diversity measures (p < 0.05 in all cases; Table 3). However, marginal r^2^ values were consistently low, showing a relatively low amount of variance explained by the relationship of each pair of diversity measures (range 0.022 to 0.387; Table 3).

**Table 3:** Triangular matrix indicating correlations of five diversity measure values among grid cells.

		Total	Threatened	Endemic	Sensitive
Threatened	slope	0.252			
	t value	8.781***			
	marginal r^2^	0.064			
	Moran's I	0.108***			
Endemic	slope	0.305	0.516		
	t value	7.329***	7.798***		
	marginal r^2^	0.045	0.180		
	Moran's I	0.043*	0.097***		
Sensitive	slope	0.209	0.360	0.415	
	t value	6.234***	7.625***	6.901***	
	marginal r^2^	0.033	0.167	0.218	
	Moran's I	0.075***	0.089***	0.032*	
T+D+LH	slope	0.182	0.640	0.640	0.686
	t value	2.047*	4.570***	2.967**	6.177***
	marginal r^2^	0.022	0.190	0.193	0.387
	Moran's I	0.070***	0.033*	0.020**	0.050**

Total, threatened, endemic and sensitive species richness are count-based diversity measures, whereas T+D+LH is a scoring method which takes into account a species threat status (T), distribution (D) and life history (LH) and assigns each grid cell an aggregate score on the basis of the species which are known to occur within that grid cell. Slope, t-value and marginal r^2^ values were calculated from spatial generalized linear mixed effects models.

* p < 0.05

** p < 0.01

*** p <0.001

**Fig 3:**
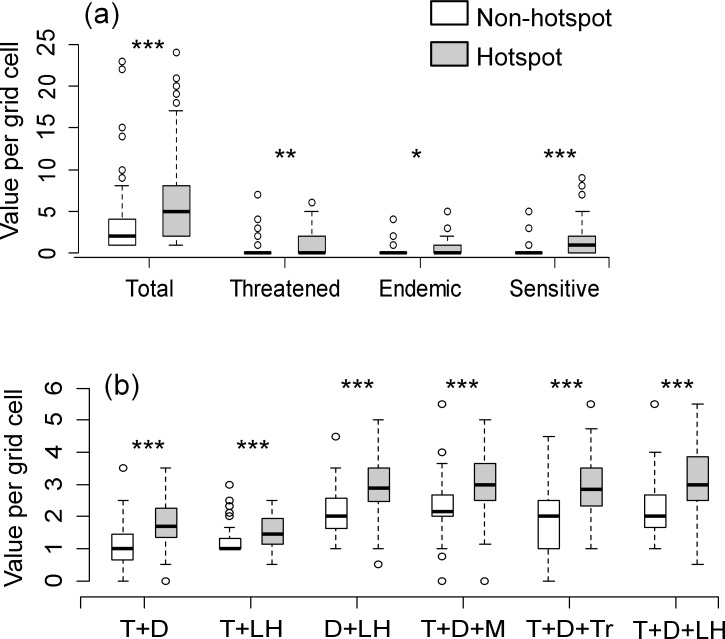
Diversity measure comparison among hotspots vs. non-hotspots. Box and whisper plots comparing median count-based (a) and score-based (b) diversity measure in biodiversity hotspot vs. non-hotspot grid cells. Diversity measure scores were calculated as described in Table 1. Mann-Whitney non-parametric tests were used to assess differences in values. T = threat status; D = distribution; M = mobility; Tr = trophic level; LH = life history. Dots indicate outlying values. * p < 0.05; ** p < 0.01; *** p <0.001

Total species richness was most highly correlated with threatened species richness, but did not correlate very well with any of the count-based or score-based measures (Table 3). Threatened, endemic and sensitive species richness, however, did correlate relatively well with each other. The T+D+LH score-based measure was highly correlated with threatened, endemic and sensitive species richness. Assuming that sampling was sufficient (see Fig 2), grid cells which fell within biodiversity hotspots had significantly higher median scores for all calculated count and score-based diversity measures than non-hotspot grid cells (Fig 3).

Katydid count-based hotspots were defined as those grid cells whose value was within the top 10% for total, threatened, endemic and/or sensitive species richness and katydid score-based hotspots were within the top 10% for T+D+LH score (S3 Fig). The cutoff value of 10% was selected because this value had apparent natural cutoff points in most of the datasets (excluding sensitive species richness).

**Fig 4:**
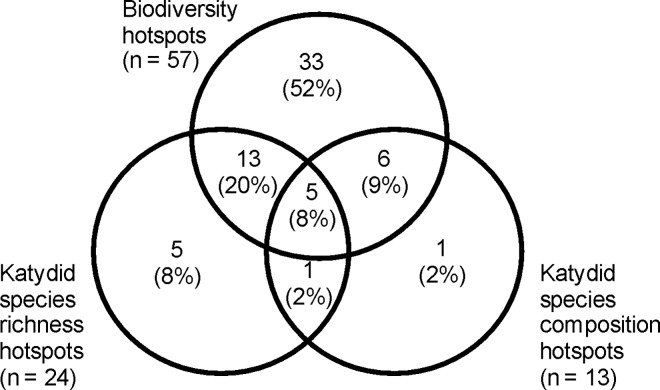
Types of hotspot distribution. Venn diagram illustrating number and percentage of grid cells selected as biodiversity hotspots, katydid species richness hotspots or katydid species composition hotspots and the degree of overlap between them.

Just over half of all grid cells (n = 64; 52%) fell within one or more of the biodiversity or katydid hotspots. Many more grid cells were classified as biodiversity hotspots than katydid hotspots (n = 57 biodiversity vs. 24 katydid count-based vs. 13 katydid score-based hotspots; Fig 4). Overlap between the three types of hotspots was large, and only five and one grid cells, respectively, were classified as only katydid count-based or katydid score-based hotspots. The rest of the grid cells were classified as hotspots under at least two of the three criteria.

Grid cells which fell within a katydid count-based or score-based hotspot were significantly more likely to also fall within a biodiversity hotspot than would be expected on the basis of chance alone (katydid count-based vs. biodiversity hotspot: χ^2^ = 9.60, p = 0.002; katydid score-based vs. biodiversity hotspot: χ^2^ = 8.39, p = 0.004). Similarly, grid cells which fell within a katydid count-based hotspot were significantly more likely to also fall within a katydid score-based hotspot than would be expected by chance alone (katydid count-based vs. score-based hotspot: χ^2^ = 6.46, p = 0.011).

**Fig 5:**
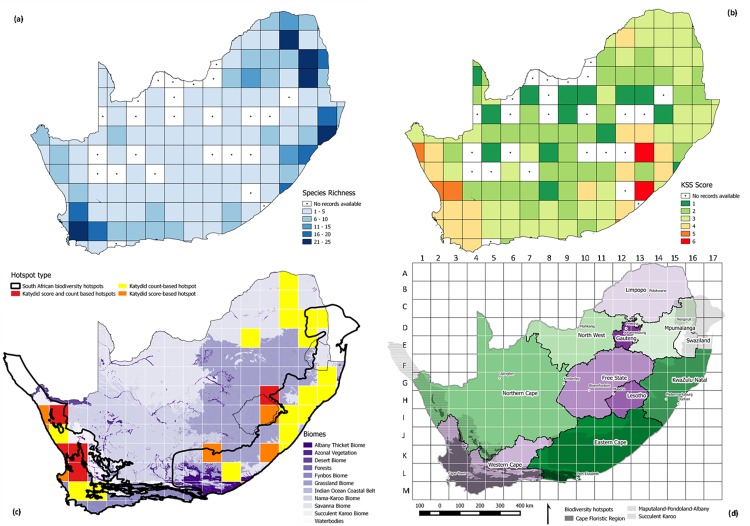
Katydid hotspot maps. Maps of katydid total species richness (a), mean T+D+LH scores (b), katydid and biodiversity hotspot locations (c), and a reference map illustrating geographic regions in South Africa, Lesotho and Swaziland (d).

Higher values of overall, threatened, and endemic species richness were found in Limpopo and along South Africa’s coastlines in the Western Cape and in KwaZulu-Natal/Eastern Cape (Fig 5A, S4 Fig). Sensitive species richness was highest in the CFR (Figure C in S4 Fig). Cells with “0” values or no available records were clustered in South Africa’s interior. Highest T+D+LH scores were found in Lesotho, Northern, Western and Eastern Cape Provinces (Fig 5B).

Six grid cells fit the criteria to be included in both count-based and score-based katydid hotspots (Fig 5C). These fell along the West Coast in the CFR and Succulent Karoo (grid cells H2, J2, J3, K3; Fig 5D), in the region of the southeastern CFR (M9) and in northern Lesotho/border of KwaZulu-Natal and Free State Provinces (G13). All but one of these grid cells fell within recognized biodiversity hotspots, and even this one grid cell did overlap with the MPA hotspot but the grid cell did not surpass the 50% inclusion rule for consideration as a “biodiversity hotspot” grid cell. The five count-based katydid hotspot grid cells which did not fall within a biodiversity hotspot were all located in Limpopo and Northwest Provinces (A15, B15, C15, E11) and the one score-based hotspot which fell outside of a biodiversity hotspot was in eastern Lesotho (H13).

## Discussion

The results of this study show clear congruence between katydid hotspots and biodiversity hotspots. In a chi-squared test we found that if a grid cell fell within either type of katydid hotspot, it was more likely to also fall within the other type of hotspot or within a biodiversity hotspot, indicating significant association between the three types of hotspots. Furthermore, values for all count-based and score-based diversity measures were significantly higher in grid cells which fell within biodiversity hotspots than in grid cells which fell outside of biodiversity hotspots. This result is not intuitive since global biodiversity hotspots were defined on the basis of vertebrate and plant diversity [1] and much ongoing debate has centered around the value of the biodiversity hotspots for the protection of invertebrates, and insects in particular [17, 19].

In order to compare congruence of katydid hotspots with recognized global biodiversity hotspots in South Africa, we first had to resolve a definition of “katydid hotspots”. Overall, threatened, and endemic species richness are all measures which have been used in the past for identifying hotspots [3, 6]. Similarly to other studies which have found little congruence among species richness count-based diversity measures [6], in a spatial GLMM we too found that correlation among overall, threatened and endemic species richness was positive and significant, but not particularly strong, and contained a large amount of unexplained variance. The relationships between endemic and overall or threatened species richness had higher slope estimates than the relationship of overall with threatened species richness, indicating that of the three count-based diversity measures, endemic species richness would be the most successful surrogate for the others.

Slope estimates for overall vs. sensitive species richness or T+D+LH, two additional diversity measures which took species biological traits into account in more detail, were the lowest of all those tested. This can best be explained by the fact that South Africa’s savanna and grassland regions, while harboring several endemic and threatened species, did not harbor many specialist herbivores of low mobility. Distinct pockets of endemic vegetation in South Africa’s biodiversity hotspots create conditions for diversification and specialization which do not exist to the same degree elsewhere in South Africa [47]. Indeed, when comparing the map of overall species richness (Fig 5A) with that of T+D+LH (Fig 5B), we see emergence of distinct hotspots entirely, with species richness hotspots located in Limpopo, KwaZulu-Natal, Eastern Cape and Western Cape Provinces, and T+D+LH hotspots located in Lesotho and elsewhere in the Northern, Western, and Eastern Cape. This pattern illustrates that high species richness does not always equate to the presence of more “valuable” species.

T+D+LH proved to be a very strong predictor for all count-based diversity measures with the exception of overall species richness in a spatial GLMM. The two principal differences between this measure and the count-based diversity measures are that: (1) its value includes fractions and ranges from 0 to 9 while the count-based diversity measures can be any whole number; and that (2) each of the count-based diversity measures, even if they take species composition into account, consider only one biological characteristic at a time while T+D+LH is a composite score which takes into account many aspects of a species’ natural history in a single value. Therefore, we conclude that species richness is the least successful of all of the surrogates, and that a score-based diversity measure like T+D+LH should be applied whenever possible since it both takes into account multiple factors of the species biology and correlates strongly with count-based diversity measures.

Comparisons of biodiversity hotspots vs. non-hotspot regions relied on the assumption that species sampling was equivalent among the regions. Species accumulation curves indicated uncertainty in this regard. The sample-based curve showed no overlap in confidence intervals and sufficient sampling in both regions, while the individual-based curve indicated overlap in the confidence intervals of the two regions. Since this is inconclusive, from experience, we expect that more sampling may have been completed along South Africa’s coastlines (where the biodiversity hotspots are located) than in the arid and inaccessible interior, but we also expect that the relatively lush and habitat-diverse coastline indeed contains greater species richness and abundance than the inhospitable interior. This issue will not be resolved until more sampling is completed and dedicated studies are designed to test this hypothesis.

Katydids are cryptic, nocturnal insects which are rarely encountered, so museum collections are small (a similar analysis on dragonflies had ten times the number of historical collection records available for analysis [12]). Additionally, biological traits and phylogenetic relationships were necessarily inferred as conservatively as possible according to expert knowledge since these data have not been collected for each individual species. Despite these sources of error, inherent differences were detected at the species level. Threatened species had significantly higher scores for distribution, mobility and life history than LC species (but not trophic level). Furthermore, in PGLS analyses, models which utilized distribution as response variable showed a significant influence of phylogeny, while those in which threat status was the response variable did not. While biological traits did conform to phylogenetic guidelines, threat status did not and threatened species were evenly distributed among all of the subfamilies included in this study.

### Recommendations and future work

The results of this study indicate that South African katydid hotspots overlap to a great degree with biodiversity hotspots. However, more dedicated sampling is necessary in order to conduct finer scale analyses of diversity patterns. The development of a score-based diversity measure (T+D+LH) holds promise for rapid monitoring of terrestrial habitats similar to the DBI for dragonflies in freshwater habitats [12, 13]. This technique is particularly exciting since katydids are acoustic animals which could be sampled in a non-invasive and non-labor intensive manner by recording of their nighttime calls, potentially allowing for assessment of areas which are difficult to sample (e.g. dense fynbos, forests or thickets). Suggested future work includes testing of T+D+LH for habitat quality assessment on a landscape-scale (as opposed to national scale as was done in this study) and comparison of results with those for dragonflies to assess the indicator potential of the katydid assemblage for another organism and for the rapid assessment of South African terrestrial habitats. Additionally, in future, distribution patterns can be correlated with environmental variables which could then be extrapolated to produce a fine-scale predictive map of katydid distribution in South Africa.

## Supporting Information

S1 Table Raw collection records data underlying the findings. Spreadsheet consisting of 1075 collection records extracted from Piotr Naskrecki’s MANTIS database. Each row represents an individual specimen record and includes taxonomic information and collecting information: country, locality description, GPS coordinates, name of collector(s), and date of collection.
(XLSX)

S2 Table South African katydid species. List of South African katydid species included in this study and their threat, distribution, mobility, trophic level and life history scores.
(DOCX)

S1 Fig Extent of occurrence distribution. Scatterplot showing that there is a natural cutoff in species distribution at extent of occurrence (EOO) = 5000 km^2^. For species with EOO < 5000 km^2^ (narrow distribution) the difference between two consecutive EOO values is a much greater proportion of the EOO value than for species with EOO > 5000 km^2^. Dashed line indicates the position of EOO = 5000 km^2^.
(TIF)

S2 Fig Phylogeny of South African katydids. Phylogenetic tree constructed for all South African Red Listed katydid species excluding data deficient (DD) species (N = 114). Branch lengths are equal to one. Subfamily relationships were assessed from Mugleston *et al*. (2013).
(DOCX)

S3 Fig Histograms illustrating katydid hotspot selection criteria. Frequency histograms showing distribution of grid cell values for total (a), threatened (b), endemic (c), and sensitive species richness (d), and T+D+LH species scores (e). Arrows indicate cutoff position for highest 10% of values. All grid cells to the right of the arrow are considered katydid hotspots.
(DOCX)

S4 Fig Supplementary maps of katydid distribution. Maps of katydid threatened (a), endemic (b), and sensitive (c) species richness.
(TIF)
