# Supplementary material for: Testing the Efficacy of Global Biodiversity Hotspots for Insect Conservation: The Case of South African Katydids
Source: PLoS One. 2016 Sep 15;11(9):e0160630. doi: 10.1371/journal.pone.0160630 (PMC5025148; doi:10.1371/journal.pone.0160630)
Supplement: S2 Table — List of South African katydid species included in this study and their threat, distribution, mobility, trophic level and life history scores. (DOCX) [file pone.0160630.s002.docx]

**S2 Table. South African katydid species.**

| Subfamily | Species | Threat Status (T) | Distribution (D) | Mobility (M) | Trophic level (Tr) | Life History (LH) |
| --- | --- | --- | --- | --- | --- | --- |
| Hetrodinae | *Acanthoplus discoidalis* | LC | 0 | 2 | 0 | 1 |
| Hetrodinae | *Acanthoplus longipes* | LC | 2 | 2 | 0 | 1 |
| Hetrodinae | *Acanthoplus speiseri* | LC | 2 | 2 | 0 | 1 |
| Hetrodinae | *Acanthoproctus cervinus* | LC | 1 | 2 | 0 | 1 |
| Hetrodinae | *Acanthoproctus diadematus* | LC | 2 | 2 | 0 | 1 |
| Hetrodinae | *Acanthoproctus vittatus* | LC | 2 | 2 | 0 | 1 |
| Pseudophyllinae | *Acauloplax exigua* | LC | 2 | 1 | 2 | 2 |
| Meconematinae | *Acilacris furcatus* | CR | 3 | 2 | 2 | 3 |
| Meconematinae | *Acilacris kristinae* | CR | 3 | 2 | 2 | 3 |
| Meconematinae | *Acilacris obovatus* | LC | 3 | 2 | 2 | 3 |
| Meconematinae | *Africariola longicauda* | VU | 3 | 2 | 2 | 3 |
| Pseudophyllinae | Gen n. sp. n. | EN | 3 | 1 | 2 | 2 |
| Tettigoniinae | *Alfredectes browni* | DD | 3 | 2 | 0 | 1 |
| Tettigoniinae | *Alfredectes semiaeneus* | LC | 3 | 2 | 0 | 1 |
| Meconematinae | *Amyttacta farrelli* | LC | 3 | 2 | 3 | 3 |
| Meconematinae | *Amyttacta marakelensis* | VU | 3 | 2 | 2 | 3 |
| Tettigoniinae | *Anarytropteris* sp. n. | VU | 3 | 2 | 0 | 1 |
| Mecopodinae | *Anoedopoda lamellata* | LC | 0 | 0 | 2 | 1 |
| Mecopodinae | *Aprosphylus olszanowskii* | LC | 3 | 0 | 2 | 1 |
| Mecopodinae | *Aprosphylus sopatarum* | DD | 3 | 0 | 2 | 1 |
| Mecopodinae | *Aprosphylus hybridus* | LC | 2 | 0 | 2 | 1 |
| Phaneropterinae | *Arantia* sp. n. | LC | 2 | 0 | 2 | 1 |
| Phaneropterinae | *Arantia fasciata* | LC | 1 | 0 | 2 | 1 |
| Tettigoniinae | *Aroegas dilatatus* | VU | 3 | 2 | 2 | 3 |
| Tettigoniinae | *Aroegas fuscus* | EN | 3 | 2 | 2 | 3 |
| Tettigoniinae | *Aroegas nigroornatus* | CR | 3 | 2 | 2 | 3 |
| Tettigoniinae | *Aroegas rentzi* | LC | 2 | 2 | 2 | 3 |
| Tettigoniinae | *Arytropteris basalis* | VU | 3 | 2 | 0 | 1 |
| Tettigoniinae | *Arytropteris granulithorax* | LC | 2 | 2 | 0 | 1 |
| Tettigoniinae | *Arytropteris pondo* | CR | 3 | 2 | 0 | 1 |
| Phaneropterinae | *Austrodontura capensis* | LC | 3 | 2 | 2 | 3 |
| Phaneropterinae | *Austrodontura castletoni* | CR | 3 | 2 | 2 | 3 |
| Phaneropterinae | *Brinckiella aptera* | VU | 3 | 2 | 3 | 3 |
| Phaneropterinae | *Brinckiella arboricola* | EN | 3 | 2 | 3 | 3 |
| Phaneropterinae | *Brinckiella elegans* | DD | 3 | 2 | 3 | 3 |
| Phaneropterinae | *Brinckiella karooensis* | VU | 3 | 2 | 3 | 3 |
| Phaneropterinae | *Brinckiella mauerbergerorum* | VU | 3 | 2 | 3 | 3 |
| Phaneropterinae | *Brinckiella serricauda* | DD | 3 | 2 | 3 | 3 |
| Phaneropterinae | *Brinckiella* sp. n. 1 | EN | 3 | 2 | 3 | 3 |
| Phaneropterinae | *Brinckiella* sp. n. 2 | CR | 3 | 2 | 3 | 3 |
| Phaneropterinae | *Brinckiella* sp. n. 3 | CR | 3 | 2 | 3 | 3 |
| Phaneropterinae | *Brinckiella viridis* | DD | 3 | 2 | 3 | 3 |
| Phaneropterinae | *Brinckiella wilsoni* | LC | 3 | 2 | 3 | 3 |
| Phaneropterinae | *Catoptropteryx aurita* | LC | 1 | 0 | 2 | 1 |
| Mecopodinae | *Cedarbergeniana imperfecta* | CR | 3 | 2 | 3 | 3 |
| Tettigoniinae | *Ceresia pulchripes* | LC | 3 | 2 | 0 | 1 |
| Tettigoniinae | *Ceresia* sp. n. | LC | 3 | 2 | 0 | 1 |
| Saginae | *Clonia (C.) kalahariensis* | LC | 2 | 0 | 1 | 1 |
| Saginae | *Clonia (C.) saussurei* | LC | 1 | 0 | 1 | 1 |
| Saginae | *Clonia (C.) uvarovi* | VU | 3 | 0 | 1 | 1 |
| Saginae | *Clonia (Hemiclonia) assimilis* | LC | 2 | 2 | 1 | 2 |
| Saginae | *Clonia (Hemiclonia) charpentieri* | DD | 3 | 2 | 1 | 2 |
| Saginae | *Clonia (Hemiclonia) lalandei* | VU | 2 | 2 | 1 | 2 |
| Saginae | *Clonia (Hemiclonia) melanoptera* | LC | 2 | 2 | 1 | 2 |
| Saginae | *Clonia (Leptoclonia) minuta* | LC | 3 | 2 | 1 | 2 |
| Saginae | *Clonia (Leptoclonia) vansoni* | LC | 3 | 2 | 1 | 2 |
| Saginae | *Clonia (Leptoclonia) vittata* | LC | 1 | 1 | 1 | 1 |
| Saginae | *Clonia (Xanthoclonia) tessellata* | LC | 2 | 2 | 1 | 2 |
| Saginae | *Clonia wahlbergi* | LC | 1 | 0 | 1 | 1 |
| Saginae | *Cloniella praedatoria* | DD | 3 | 2 | 1 | 2 |
| Phaneropterinae | *Conchotopoda belcki* | DD | 2 | 1 | 0 | 1 |
| Phaneropterinae | *Conchotopoda brunneri* | DD | 3 | 1 | 0 | 1 |
| Phaneropterinae | *Conchotopoda crassicauda* | DD | 2 | 0 | 0 | 0 |
| Phaneropterinae | *Conchotopoda grallatoria* | DD | 3 | 1 | 0 | 1 |
| Phaneropterinae | *Conchotopoda parva* | DD | 3 | 1 | 0 | 1 |
| Conocephalinae | *Conocephalus (Anisoptera) maculatus* | LC | 0 | 0 | 2 | 1 |
| Conocephalinae | *Conocephalus (C.) basutoanus* | EN | 3 | 2 | 2 | 3 |
| Conocephalinae | *Conocephalus (C.) caudalis* | LC | 1 | 2 | 0 | 1 |
| Conocephalinae | *Conocephalus (C.) conocephalus* | LC | 0 | 0 | 2 | 1 |
| Conocephalinae | *Conocephalus (C.) peringueyi* | VU | 3 | 2 | 2 | 3 |
| Conocephalinae | *Conocephalus (Xiphidion) iris* | LC | 0 | 2 | 0 | 1 |
| Conocephalinae | *Conocephalus longiceps* | LC | 2 | 2 | 2 | 3 |
| Conocephalinae | *Conocephalus zlobini* | VU | 3 | 2 | 2 | 3 |
| Phaneropterinae | *Corymeta amplectens* | LC | 2 | 0 | 2 | 1 |
| Pseudophyllinae | *Cymatomera denticollis* | LC | 1 | 1 | 2 | 2 |
| Pseudophyllinae | *Cymatomerella spilophora* | LC | 2 | 1 | 2 | 2 |
| Phaneropterinae | *Ducetia chelocerca* | DD | 3 | 0 | 2 | 1 |
| Hetrodinae | *Enyaliopsis transvaalensis* | LC | 2 | 2 | 0 | 1 |
| Phaneropterinae | *Eulioptera flexilima* | LC | 1 | 0 | 2 | 1 |
| Phaneropterinae | *Eulioptera spinulosa* | LC | 0 | 0 | 2 | 1 |
| Phaneropterinae | *Eulioptera reticulata* | LC | 0 | 0 | 2 | 1 |
| Phaneropterinae | *Eurycorypha cereris* | LC | 2 | 0 | 2 | 1 |
| Phaneropterinae | *Eurycorypha lesnei* | LC | 2 | 0 | 2 | 1 |
| Phaneropterinae | *Eurycorypha meruensis* | LC | 3 | 0 | 2 | 1 |
| Phaneropterinae | *Eurycorypha proserpinae* | LC | 3 | 0 | 2 | 1 |
| Mecopodinae | *Griffiniana capensis* | LC | 2 | 2 | 2 | 3 |
| Mecopodinae | *Griffiniana duplessisae* | CR | 3 | 1 | 2 | 2 |
| Mecopodinae | *Griffiniana longipes* | LC | 2 | 0 | 2 | 1 |
| Hetrodinae | *Hemihetrodes bachmanni* | LC | 2 | 2 | 0 | 1 |
| Hetrodinae | *Hetrodes pupus* | LC | 2 | 2 | 0 | 1 |
| Phaneropterinae | *Horatosphaga serrifera* | LC | 0 | 0 | 0 | 0 |
| Conocephalinae | *Lanista annulicornis* | LC | 0 | 0 | 3 | 2 |
| Conocephalinae | *Megalotheca montana* | LC | 3 | 2 | 3 | 3 |
| Conocephalinae | *Megalotheca vaginalis* | EN | 3 | 2 | 3 | 3 |
| Phaneropterinae | *Melidia brunneri* | LC | 1 | 0 | 2 | 1 |
| Tettigoniinae | *Namaquadectes irroratus* | DD | 3 | 2 | 0 | 1 |
| Phaneropterinae | *Oxyecous lesnei* | LC | 2 | 0 | 2 | 1 |
| Meconematinae | *Paracilacris lateralis* | VU | 2 | 2 | 2 | 3 |
| Meconematinae | *Paracilacris mordax* | VU | 3 | 2 | 2 | 3 |
| Meconematinae | *Paracilacris periclitatus* | CR | 3 | 2 | 2 | 3 |
| Meconematinae | *Paracilacris* sp. n. | LC | 3 | 2 | 2 | 3 |
| Saginae | *Peringueyella rentzi* | EN | 3 | 2 | 1 | 2 |
| Saginae | *Peringueyella zulu* | CR | 3 | 2 | 1 | 2 |
| Saginae | *Peringueyella jocosa* | LC | 1 | 2 | 1 | 2 |
| Phaneropterinae | *Phaneroptera gracilis* | LC | 0 | 0 | 2 | 1 |
| Phaneropterinae | *Phaneroptera nigropunctata* | DD | 2 | 0 | 2 | 1 |
| Phaneropterinae | *Phaneroptera sparsa* | LC | 0 | 0 | 2 | 1 |
| Phaneropterinae | *Plangia compressa* | LC | 2 | 0 | 2 | 1 |
| Phaneropterinae | *Plangia graminea* | LC | 1 | 0 | 2 | 1 |
| Mecopodinae | *Pomatonota dregii* | VU | 3 | 0 | 2 | 1 |
| Phaneropterinae | *Prosphaga calaharica* | LC | 2 | 1 | 0 | 1 |
| Conocephalinae | *Pseudorhynchus hastifer* | LC | 0 | 0 | 2 | 1 |
| Conocephalinae | *Pseudorhynchus pungens* | LC | 0 | 0 | 2 | 1 |
| Mecopodinae | *Pseudosaga maculata* | LC | 2 | 0 | 2 | 1 |
| Conocephalinae | *Ruspolia ampla* | LC | 0 | 0 | 0 | 0 |
| Phaneropterinae | *Symmetropleura plana* | DD | 3 | 0 | 2 | 1 |
| Phaneropterinae | *Terpnistria lobulata* | LC | 2 | 0 | 2 | 1 |
| Phaneropterinae | *Terpnistria zebrata* | LC | 1 | 0 | 2 | 1 |
| Tettigoniinae | *Thoracistus arboreus* | CR | 3 | 2 | 0 | 1 |
| Tettigoniinae | *Thoracistus aureoportalis* | VU | 3 | 2 | 0 | 1 |
| Tettigoniinae | *Thoracistus jambila* | EN | 3 | 2 | 0 | 1 |
| Tettigoniinae | *Thoracistus peringueyi* | CR | 3 | 2 | 0 | 1 |
| Tettigoniinae | *Thoracistus semeniphagus* | EN | 3 | 2 | 0 | 1 |
| Tettigoniinae | *Thoracistus thyraeus* | EN | 3 | 2 | 0 | 1 |
| Tettigoniinae | *Thoracistus viridicrus* | VU | 3 | 2 | 0 | 1 |
| Tettigoniinae | *Thoracistus viridifer* | LC | 3 | 2 | 0 | 1 |
| Tettigoniinae | *Transkeidectes multidentis* | CR | 3 | 2 | 0 | 1 |
| Phaneropterinae | *Tylopsis bilineolata* | LC | 0 | 0 | 2 | 1 |
| Phaneropterinae | *Tylopsis continua* | LC | 0 | 0 | 2 | 1 |
| Phaneropterinae | *Tylopsis rubrescens* | LC | 0 | 0 | 2 | 1 |
| Pseudophyllinae | *Zabalius ophthalmicus* | LC | 1 | 1 | 2 | 2 |
| Mecopodinae | *Zitsikama tesselata* | LC | 3 | 2 | 2 | 3 |
| Tettigoniinae | *Zuludectes modestus* | LC | 1 | 2 | 0 | 1 |
