# Supplementary figures and images for: Testing the Efficacy of Global Biodiversity Hotspots for Insect Conservation: The Case of South African Katydids
Source: PLoS One. 2016 Sep 15;11(9):e0160630. doi: 10.1371/journal.pone.0160630 (PMC5025148; doi:10.1371/journal.pone.0160630)

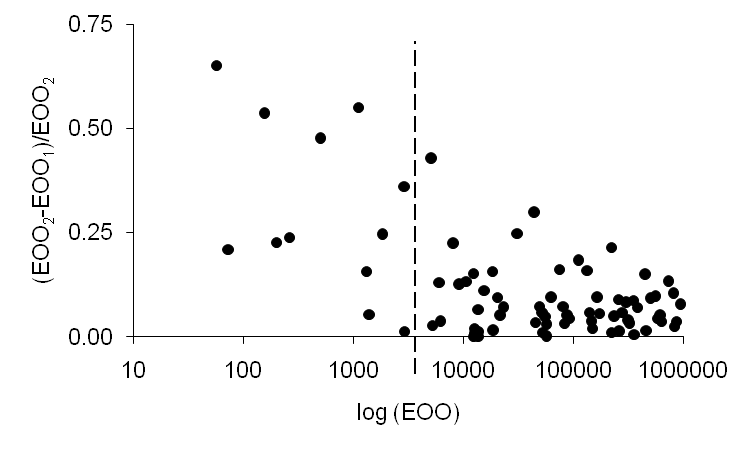

Supplement: S1 Fig — Scatterplot showing that there is a natural cutoff in species distribution at extent of occurrence (EOO) = 5000 km2. For species with EOO < 5000 km2 (narrow distribution) the difference between two consecutive EOO values is a much greater proportion of the EOO value than for species with EOO > 5000 km2. Dashed line indicates the position of EOO = 5000 km2. (TIF) [file pone.0160630.s003.tif]

**S2 Fig. Phylogeny of South African katydids.**
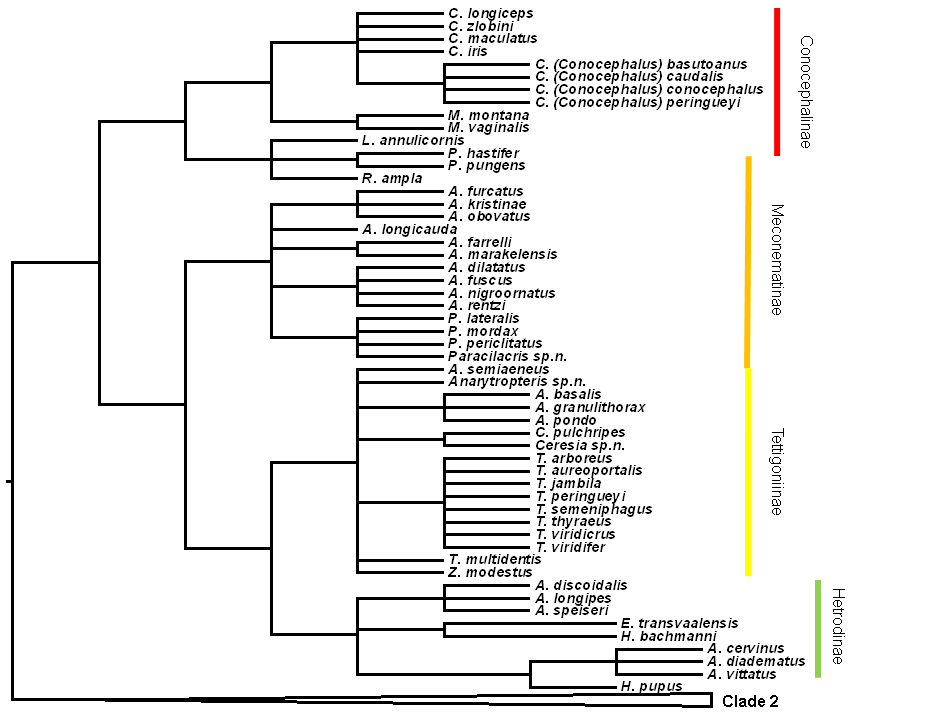


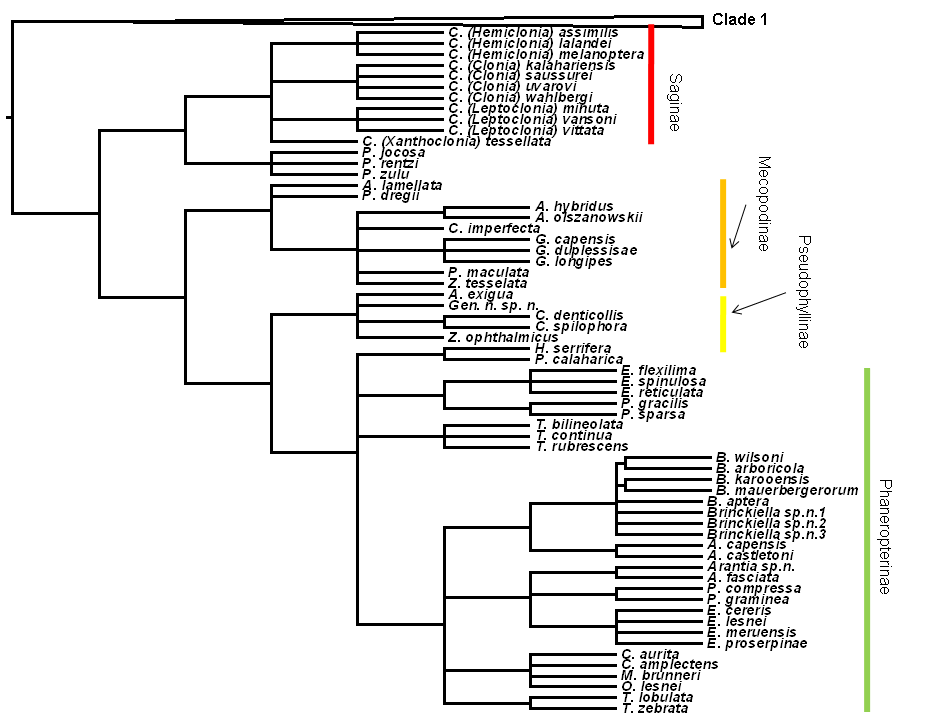

Supplement: S2 Fig — Phylogenetic tree constructed for all South African Red Listed katydid species excluding data deficient (DD) species (N = 114). Branch lengths are equal to one. Subfamily relationships were assessed from Mugleston et al. (2013). (DOCX) [file pone.0160630.s004.docx]

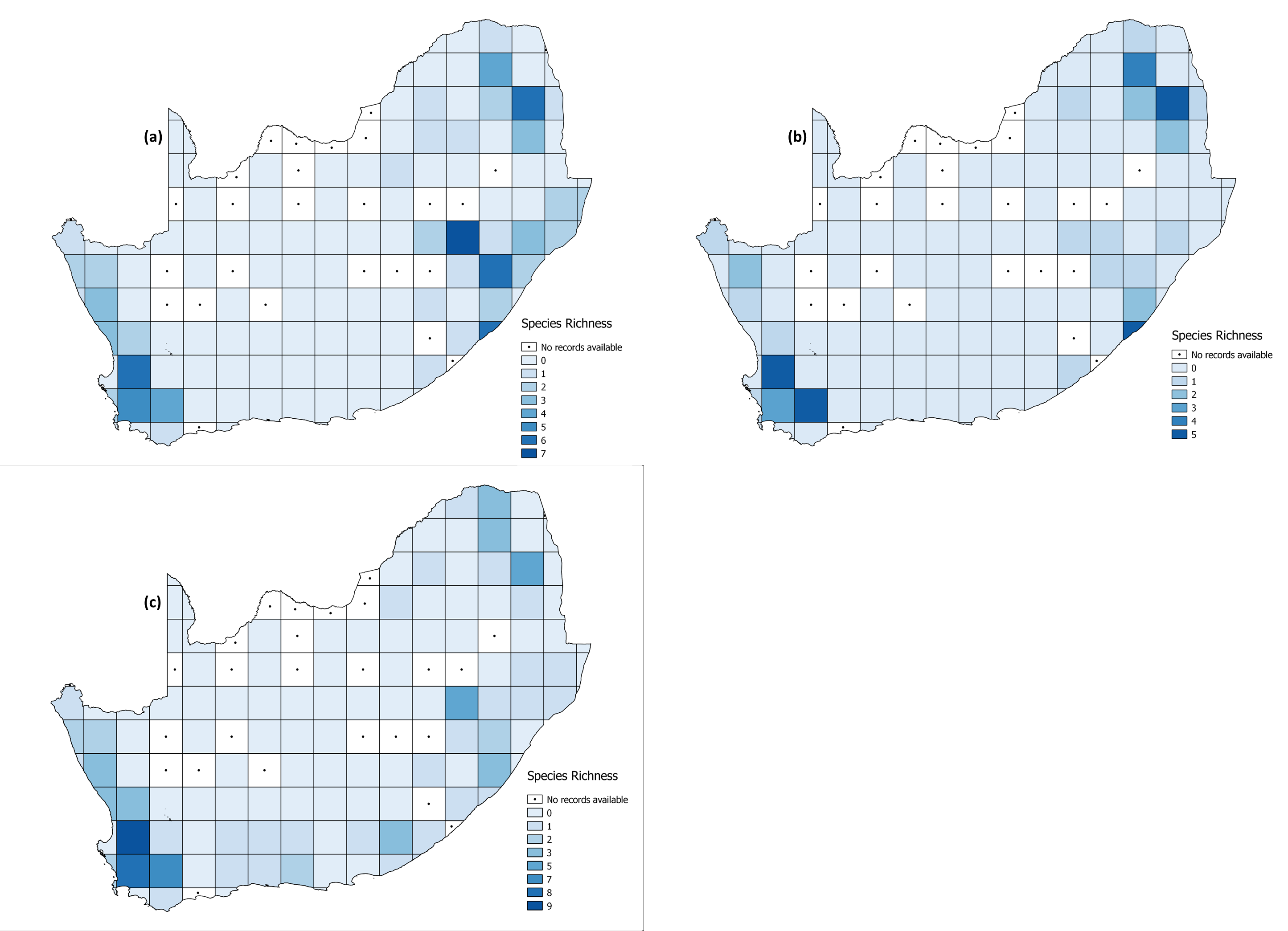

Supplement: S4 Fig — Maps of katydid threatened (a), endemic (b), and sensitive (c) species richness. (TIF) [file pone.0160630.s006.tif]
