# Supplementary material for: Testing the Efficacy of Global Biodiversity Hotspots for Insect Conservation: The Case of South African Katydids
Source: PLoS One. 2016 Sep 15;11(9):e0160630. doi: 10.1371/journal.pone.0160630 (PMC5025148; doi:10.1371/journal.pone.0160630)
Supplement: S3 Fig — Frequency histograms showing distribution of grid cell values for total (a), threatened (b), endemic (c), and sensitive species richness (d), and T+D+LH species scores (e). Arrows indicate cutoff position for highest 10% of values. All grid cells to the right of the arrow are considered katydid hotspots. (DOCX) [file pone.0160630.s005.docx]

**S3 Fig. Histograms illustrating katydid hotspot selection criteria.**
